# Suppurative minor salivary gland sialolithiasis

**DOI:** 10.4322/acr.2021.397

**Published:** 2022-09-12

**Authors:** Camila de Oliveira Barbeiro, Roberto Henrique Barbeiro, Andreia Bufalino, Jorge Esquiche León

**Affiliations:** 1 Universidade Estadual Paulista “Júlio de Mesquita Filho” (Unesp), Faculdade de Odontologia de Araraquara, Departamento de Diagnóstico e Cirurgia, Araraquara, SP, Brasil; 2 Universidade de São Paulo (USP), Faculdade de Odontologia de Ribeirão Preto, Departamento de Estomatologia, Saúde Coletiva e Odontologia Legal, Ribeirão Preto, SP, Brasil

**Keywords:** Salivary gland calculi, Sialadenitis, Salivary Glands, Minor

## Abstract

Sialolithiasis is a common nonneoplastic disease of the major salivary glands that often affects the submandibular glands. Minor salivary gland involvement by sialolithiasis is uncommon, with only 273 cases reported. A long clinical history, acute symptoms, and mucopurulent discharge are unusual features of these cases. Herein, we report the case of a 63-year-old woman who complained of symptomatic nodular swelling of the buccal mucosa associated with purulent discharge for several days. The clinical history lasted 15 years, with episodes of asymptomatic non-suppurative swelling in the same area. The patient underwent surgical excision. The microscopic examination revealed chronic nonspecific sialadenitis associated with psammomatous calcifications, confirming minor salivary gland sialolithiasis. After 3 years of follow-up, the patient was free of symptoms. Patients with sialolithiasis are usually asymptomatic; however, swelling, pain, and fistula may be present in rare cases. The presence of purulent exudate should lead to the differential diagnosis of stomatitis glandularis, a rare inflammatory condition affecting the minor salivary glands. Sialolithiasis and stomatitis glandularis should be considered in the clinical differential diagnosis of symptomatic suppurative nodular swelling affecting the oral mucosa, and histopathological analysis is necessary for the diagnosis.

## INTRODUCTION

Salivary gland disorders are of inflammatory, infectious, and neoplastic origins, and their clinical presentation can be acute or chronic. Recurrent or chronic sialadenitis is more likely to be inflammatory, as observed in recurrent childhood parotitis and sialolithiasis. Salivary gland inflammation is caused by salivary duct obstruction due to lithiasis or salivary duct stricture.^1^ Acute suppurative sialadenitis is commonly infectious and is characterized by a rapid-onset, painful swelling often affecting the parotid gland.^1^^-^2 Sialolithiasis is a common disease of the salivary glands that can cause duct obstruction, inflammation, and infection. It usually affects the major salivary glands; however, minor salivary gland involvement rarely occurs. Clinically, sialolithiasis of the minor salivary glands (SMSG) is characterized by a local and well-circumscribed bulging not detectable during radiographic examination.^1^^-^3

In 1965, Papin^4^ reported the first case of SMSG. Since then, at least 273 cases have been reported.^5^ Most SMSG cases comprise old individuals (mean age: 55 years) with upper lip and buccal mucosa involvement. In addition, a slight male predominance was observed in previous case series studies, except for the study conducted by Wen-Chen Wang, which reported a male predominance of 82%.^6^ As SMSG lacks specific clinical features, it is often asymptomatic and rarely detectable on imaging examinations. Surgical excision followed by histopathological analysis is the optimal diagnostic approach. The histopathological examination shows heterogeneous lamellated calculi within the lumen of a dilated minor salivary duct with periductal inflammation.^1^^-^8

The present case report aimed to describe an unusual case of a patient with SMSG on the buccal mucosa, which exhibited purulent drainage and pain after 15 years of evolution.

## CASE REPORT

**Figure 1:**
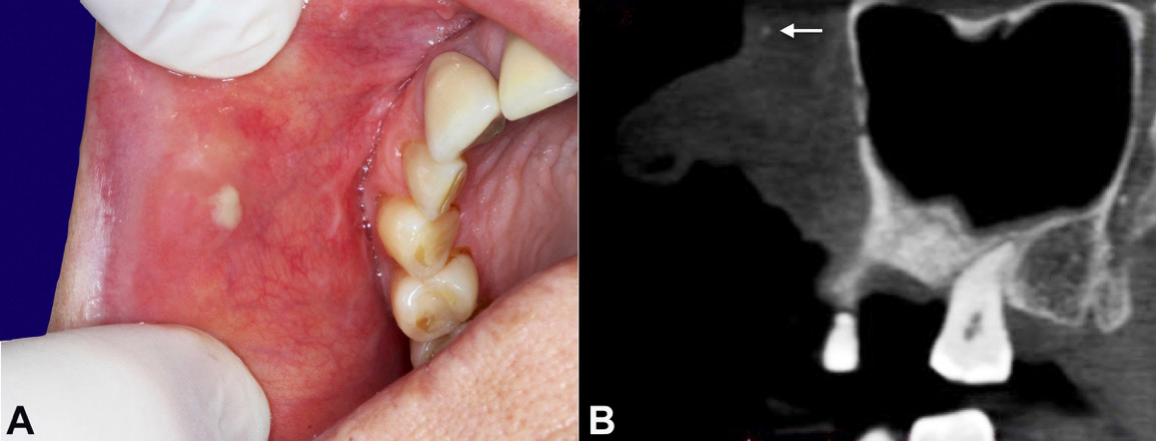
**A** - gross view of the buccal mucosa showing the nodular swelling with mucopurulent discharge; **B** - sagittal computed tomography showing a tiny hyperdense structure in the lesional area (white arrow).

A 63-year-old woman presented to the oral and maxillofacial department complaining of a painful suppurative lesion on the buccal mucosa for several days. The patient reported several episodes of asymptomatic, non-suppurative swelling in the same lesion area over the last 15 years. The patient’s medical history was unremarkable. Intraoral examination revealed a painful nodular swelling of the buccal mucosa with mucopurulent discharge (Figure 1A). The computed tomography finding (Figure 1B) was consistent with a small calcification in the buccal mucosa. After antibiotic treatment, an excisional biopsy was performed.

**Figure 2:**
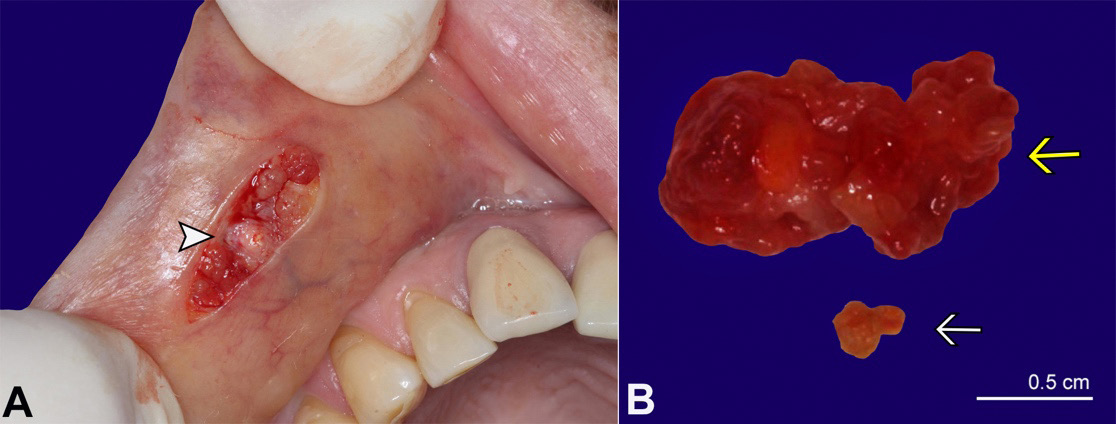
**A** - gross view of the surgical field enabling the visualization of the sialolith (arrowhead); **B** - excised specimens. The yellow arrow shows the solid mass with a smooth surface, while the white arrow shows the sialolith measuring 0.3 × 0.2 × 0.1 cm.

During surgery, a spherical, whitish calcified mass compatible with sialoliths was depicted (Figure 2A). Grossly, the hard tissue fragment measured 0.3 × 0.2 × 0.1 cm immersed in soft tissue (Figure 2B).

**Figure 3:**
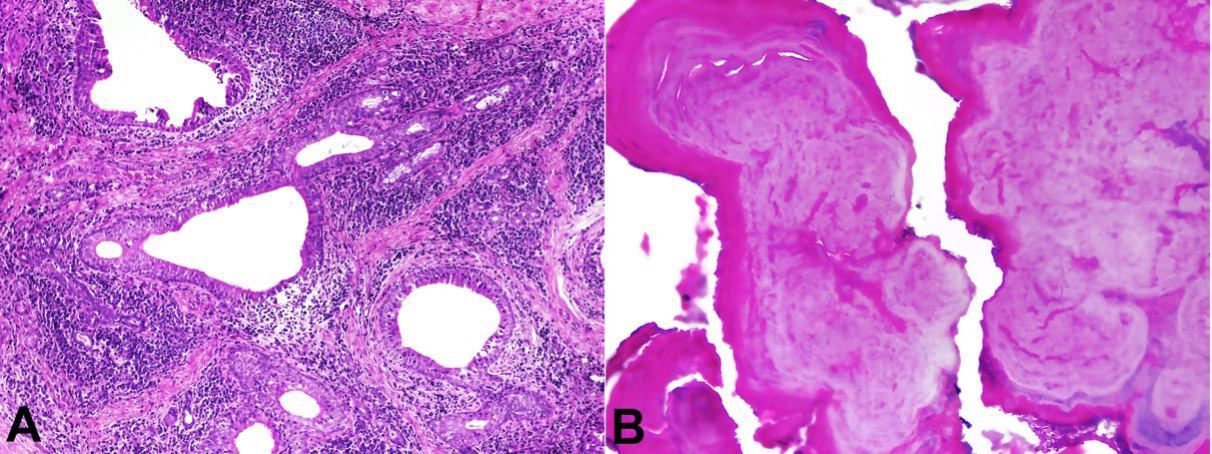
**A** - photomicrographs of the surgical specimen exhibiting fibrous stroma, acinar atrophy, and dilated ducts surrounded by chronic inflammation (H&E stain, ×10); **B** - histopathological features showing a homogeneous calcified structure in a concentric pattern (H&E stain, ×10).

The microscopic examination revealed a fibrovascular stroma containing foci of neutrophil infiltration, acinar atrophy, and numerous dilated salivary gland ducts surrounded by an abundant chronic inflammatory infiltrate (Figure 3A). The hard tissue showed basophilic calcified material with a fingerprint-like appearance in a concentric pattern (Figure 3B). The histopathological features and presence of sialoliths led to a final diagnosis of SMSG. After a 3-year follow-up, the patient had a stable condition, without recurrence or alteration in the lesion area.

## DISCUSSION

Sialolithiasis is a common salivary gland disease that occurs more frequently in middle-aged patients with a slight male predominance. Sialolithiasis commonly affects the submandibular gland (80%), followed by the parotid gland (6%–20%); sublingual and minor salivary gland involvement rarely occurs (2%).^3^ Patients with sialolithiasis typically present with postprandial salivary gland pain and swelling; some patients report recurrent acute suppurative inflammation. Although the latter is often observed in patients with major salivary gland involvement^1^^-^3, it occurred in the current case.

The SMSG usually presents as a palpable, submucosal nodule and is often asymptomatic; and mostly involves patients with a mean age of 55 years and men. The upper lip and buccal mucosa are the most affected sites, followed by the lower lip and mucobuccal folds. The signs and symptoms depend on the size and location of the sialoliths. The time of symptoms varies from several months to 3 years, mostly detected within the first year. In some cases, the lesion compression may render a purulent discharge. In the present case, symptomatic nodular swelling with mucopurulent discharge was detected after 15 years. These clinicopathological features are not common in patients with sialolithiasis. They should be distinguished from acute suppurative sialadenitis, a disease caused by bacterial infection, commonly *Staphylococcus aureus,* mainly affecting the parotid glands.^3^^-^8 Relevantly, no study has examined patients with acute suppurative sialadenitis affecting the minor salivary glands.

Treatment of sialolithiasis depends on the symptoms and the size and localization of sialoliths. Although some sialoliths are removed by manipulation of the salivary gland, most patients require surgical excision, as shown in our case. Another treatment option is lithotripsy during sialendoscopy.^1^^-^10

Mucoceles, mucous retention cysts, and salivary gland neoplasms were initially considered in the clinical differential diagnosis when assessing nodular swelling of the oral mucosa. After performing a histopathological analysis, the final diagnosis was sialolithiasis.^1^^-^9 Moreover, in the current case, stomatitis glandularis (SG) should also be considered in the differential diagnosis. This condition is an uncommon suppurative chronic inflammatory disease of the minor salivary glands that mainly affects the lips, followed by the buccal mucosa and palate. SG has three clinical types: simple, superficial suppurative, and deep suppurative. Its etiology is unclear, but it has been associated with deep infection by bacterial colonization. To date, only five cases of SG have been reported. Three patients were women, and two were men, with a mean age of 55. These patients complained of swelling and purulent exudates, which were frequently symptomatic. The time of evolution of the lesions varied from 5 to 30 years; in three cases, multiple sites were involved (upper lip, lower lip, and buccal mucosa). After surgical removal of the lesions, microscopic examination showed chronic inflammatory cell infiltration, ductal ectasia, acinar atrophy, and absence of calcification deposits.^11^^-^14

Although challenging, SMSG should be considered in the clinical differential diagnosis of suppurative nodular swelling affecting the oral mucosa. Suppuration is possible in patients with sialolithiasis of the minor salivary glands, as reported in this clinical case with a 15-year duration.
